# Isolated non-traumatic, non-aneurysmal convexal subarachnoid hemorrhage in a patient with Evans syndrome

**DOI:** 10.1186/s12883-017-0944-9

**Published:** 2017-08-25

**Authors:** Anna Misyail Abdul Rashid, Mohamad Syafeeq Faeez Md Noh

**Affiliations:** 1 Department of Medicine, Faculty of Medicine and Health Sciences, Universiti Putra, Serdang, Malaysia; 2 Department of Imaging, Faculty of Medicine and Health Sciences, Universiti Putra Malaysia, Level 3, 43400 Serdang, Selangor Malaysia

**Keywords:** Evans syndrome, Subarachnoid haemorrhage (SAH)

## Abstract

**Background:**

Non-traumatic, spontaneous subarachnoid hemorrhage occurs in approximately 85% of cases where there is a ruptured saccular aneurysm. An additional 10% of cases arise from non-aneurysmal peri-mesencephalic hemorrhages.

**Case presentation:**

We report a rare case of a young female, with underlying Evans syndrome, who was initially thought to have non-hemorrhagic stroke, eventually diagnosed having isolated non-traumatic, non-aneurysmal convexal subarachnoid haemorrhage.

**Conclusions:**

Spontaneous non-traumatic, non-aneurysmal convexal subarachnoid hemorrhage is a rare entity - of which there are multiple possible etiologies.

## Background

Classically, non-traumatic subarachnoid hemorrhage (SAH) is known to occur mostly in association with underlying aneurysm [1]. The hemorrhage is seen, whether via computed tomography (CT) or magnetic resonance imaging (MRI), in the basal cisterns. Spontaneous SAH occurring in the convexal region of the brain is a rare entity. Multiple etiologies have been attributed – these include vascular and non-vascular causes [2]. We describe a young female presenting to our center, with underlying Evans syndrome, initially misdiagnosed as suffering from acute non-hemorrhagic stroke; only to be eventually diagnosed with an acute isolated non-traumatic, non-aneurysmal convexal SAH via MRI and diagnostic cerebral angiography.

## Case presentation

**Fig. 1:**
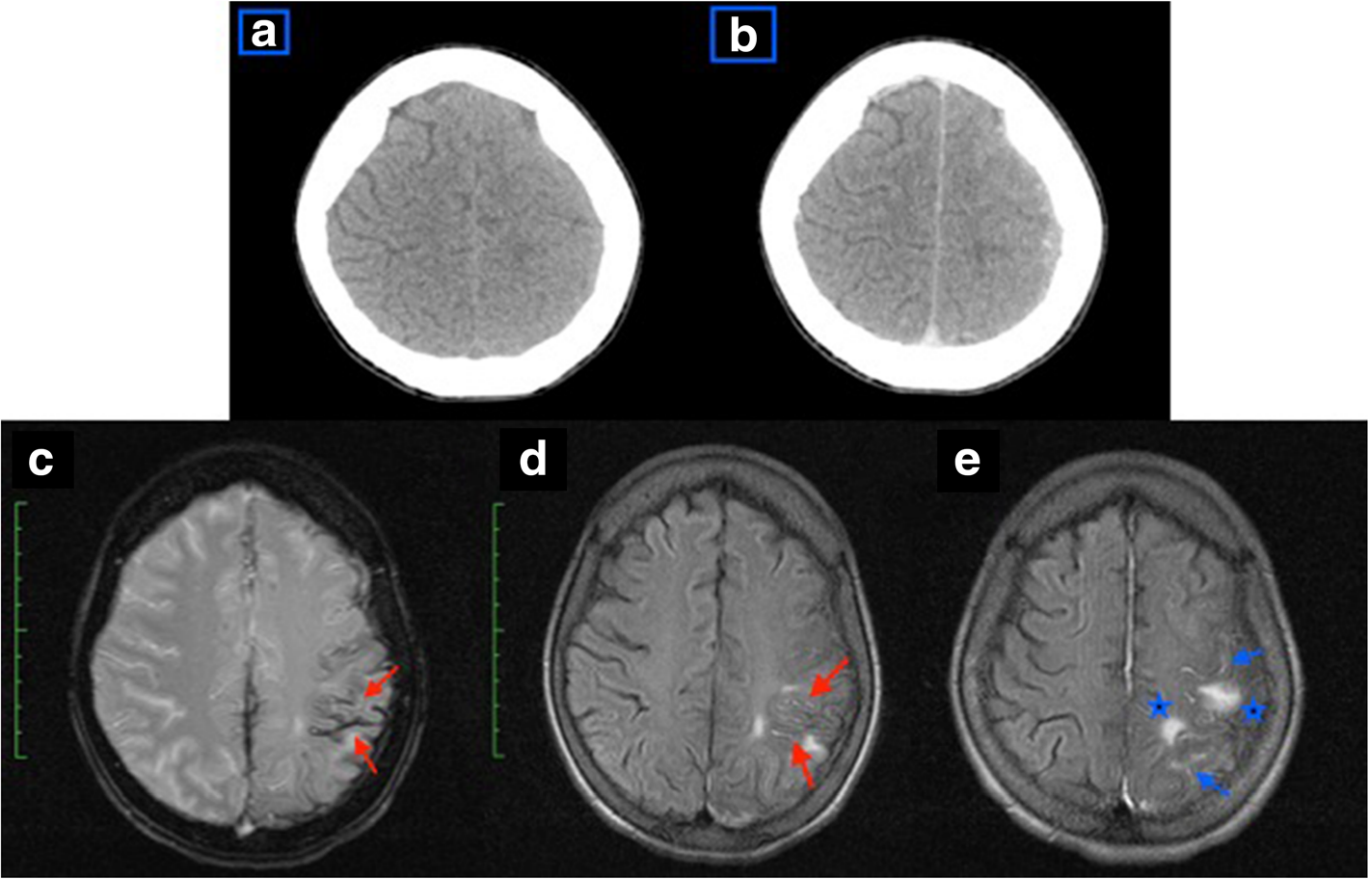
Axial CT images at the high parietal region, plain (1**a**) and contrasted (1**b**) showing; 1**a** – left high parietal region cerebral edema is present, with no evidence of haemorrhage. 1**b** – Contrast is seen opacifying the sulci, but otherwise no additional findings were evident. Axial MRI images; GRE (1**c**), pre-contrast FLAIR (1**d**), and T2 FLAIR post gadolinium (1**e**), sequences at the high parietal region showing; 1**c** – Hypointense signal of the affected region (arrows) with corresponding hyperintensity on FLAIR (1**d**) confirming acute convexal SAH. 1**e** – Areas of pachy- and leptomeningeal enhancement, with small foci of haemorrhage; indirect signs of vasculitis

**Fig. 2:**
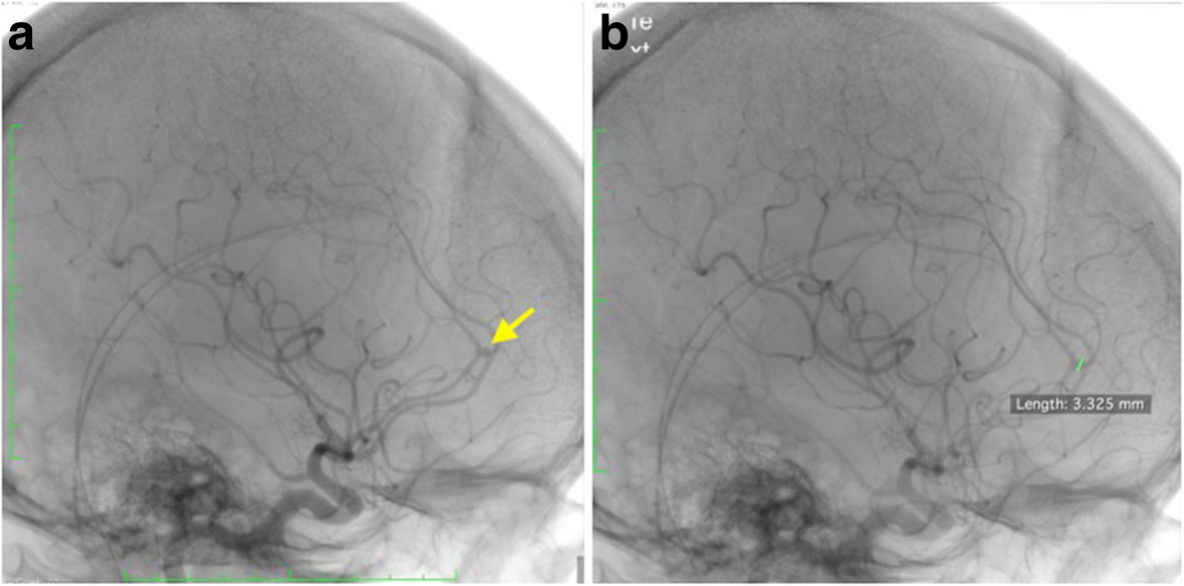
Diagnostic cerebral angiography images (2**a**, 2**b**) showing; 2**a** – the left sided non-hemorrhaging A3 aneurysm. 2**b** – The aneurysm measured 3.3 mm in its widest diameter, with no other features to suggest haemorrhage

A 39-years-old lady, with underlying Evans syndrome under Hematology follow up presented to our center with acute onset right sided hemiparesis, associated with facial asymmetry. On examination, she was afebrile. Neurological assessment revealed a power of 3/5, with brisk reflexes. Sensation was reduced on the affected side. There were no ocular manifestations. Cerebellar signs were absent. Blood investigations, including coagulation profile, were unremarkable except for slight thrombocytopenia of 120 × 10^9^/L (normal range 150–400 × 10^9^/L). Urgent plain brain CT, done a few hours after symptoms onset, revealed left high parietal region cerebral edema (HU of 50–70), with no evidence of hemorrhage (Fig. 1A). A contrasted study done the following day revealed no additional findings (Fig. 1B). A decision was made to pursue MRI, 2 days post symptom onset, due to the non-specific findings on CT. Left high parietal region low signal intensities on gradient echo (GRE) sequence, with corresponding high signal intensities seen on pre-contrast fluid-attenuated inversion recovery (FLAIR) sequence confirmed an acute SAH (Fig. 1C, D). Contrasted T2 FLAIR sequence showed accompanying lepto- and pachymeningeal enhancement (Fig. 1E). Additionally, a few small intra-parenchymal acute hemorrhages were also noted. MR angiography (MRA) and MR venography (MRV) were negative. A diagnostic cerebral angiography was pursued the following 2 weeks, despite the MRA being negative. This revealed a left sided A3 aneurysm, measuring approximately 3.3 mm in its widest dimension (Fig. 2A, B), with no features to suggest hemorrhage. The location of this aneurysm did not correspond to the region of the focal convexal subarachnoid hemorrhage – and concluded to be an incidental finding. Patient was treated conservatively, and gradually improved with steroid therapy.

## Discussion and conclusions

We report a rare case of a spontaneous isolated, non-traumatic, non-aneurysmal SAH in a young lady with underlying Evans syndrome – which is a rare haematological disorder of unknown frequency characterized by simultaneous or sequential development of autoimmune hemolytic anemia (AIHA) and immune thrombocytopenia (ITP), with or without immune neutropenia [3]**.** She was initially diagnosed 5-year prior the current presentation, when she came with complaints of intermittent, episodic bruising and bleeding, with anemic symptoms. Coombs test, and immunoglobulin G (IgG) done in a different center, was positive. Bone marrow aspiration was not pursued.

Initially described in 1951, Evans syndrome is a rare clinical entity; occurring in 0.8% to 3.7% of all patients diagnosed with ITP or AIHA at first presentation. This disorder is associated with conditions such as systemic lupus erythematosus (SLE), lymphoproliferative disorders, and primary immune-deficiencies. Patients are at increased risk of bleeding manifestations, and the first line management is steroid therapy, followed by second line pharmacotherapy, rituximab, or splenectomy; when patients do not respond to steroid therapy [3].

Isolated acute non-traumatic convexal SAH are known to be caused by multiple etiological factors; whether vascular (i.e. venous thrombosis, vasculitides) or non-vascular (i.e. abscess, tumors) [1]. Kumar et al. retrospectively reviewed 29 consecutive patients with non-traumatic convexal SAH, and concluded that amyloid angiopathy was frequent in patients aged over 60-years-old, whereas in those younger, a reversible vasoconstriction syndrome is a common cause [4].

Convexal SAH, if present, may appear as an area of slight sulcal hyperattenuation. However, in circumstances where the CT findings are barely visible or non-specific, MRI is a better option. Useful sequences would include FLAIR, GRE T2, diffusion weighted imaging (DWI), 3D time of flight (TOF) MRA, contrast enhanced venography, and pre- as well as post-gadolinium T1-weighted imaging [2]. We were only able to visualize left high parietal cerebral edema on plain and contrasted CT; thus we proceeded with the above mentioned sequences on our MRI examination.

Interestingly, this revealed an acute SAH at the involved region with small foci of intraparenchymal haemorrhage; manifesting as hyperintensity on FLAIR sequence with corresponding hypointensity on GRE sequence, and accompanying lepto- and pachymeningeal enhancement – which are indirect features of vasculitis. Michel et al. [3] in their study inferred that immune thrombocytopenia occurring in the setting of Evans syndrome may lead to bleeding manifestations. A single predictive factor, eg, thrombocytopenia poorly correlates with bleeding risk in ITP; as this is likely multifactorial [5]. Taking into account the patient’s underlying Evans syndrome, as well as indirect features of vasculitis on MRI, we concurred that in our case, MRI finding of isolated convexal SAH was likely the complication [6]. Confirmatory biopsy was not pursued as patient was not keen.

Our experience with this case portrays the additional value of MRI compared to CT; especially when dealing with rare, equivocal cases. However, due to the relative superiority of diagnostic cerebral angiography compared to MRA in detecting small intracerebral aneurysms [7], we decided to still pursue the examination, despite MRA being negative – which revealed an incidental finding of a non-hemorrhaging left sided A3 aneurysm. This strengthened our suspicion – Evans syndrome, as the likely etiology.

Isolated acute non-traumatic convexal SAH is a rare entity, with multiple possible etiological factors. Ruptured aneurysms, being the most common cause of SAH, needs to be excluded as part of the diagnostic workup. The presence of a rare disorder, such as seen in our case, complicates patient management. MRI with select sequences, complemented by diagnostic cerebral angiography prove to be powerful tools in assessment and diagnosis of equivocal cases – thus enabling proper patient management and treatment.

## Abbreviations

AIHA: Autoimmune haemolytic anemia
CT: Computed tomography
DWI: Diffusion weighted imaging
FLAIR: Fluid-attenuated inversion recovery
GRE: Gradient echo
ITP: Immune thrombocytopenia
MRA: Magnetic resonance angiography
MRI: Magnetic resonance imaging
MRV: Magnetic resonance venography
SAH: Subarachnoid haemorrhage
SLE: Systemic lupus erythematosus
TOF: Time of flight
